# Motion-Induced Position Shifts Activate Early Visual Cortex

**DOI:** 10.3389/fnins.2017.00168

**Published:** 2017-04-03

**Authors:** Peter J. Kohler, Patrick Cavanagh, Peter U. Tse

**Affiliations:** ^1^Department of Psychology, Stanford UniversityStanford, CA, USA; ^2^Laboratoire Psychologie de la Perception, Centre Biomédical des Saints Pères, Université Paris DescartesParis, France; ^3^Department of Psychological and Brain Sciences, Dartmouth CollegeHanover, NH, USA

**Keywords:** spatial vision, position perception, striate and extrastriate cortex, functional MRI, motion-induced position shifts

## Abstract

The ability to correctly determine the position of objects in space is a fundamental task of the visual system. The perceived position of briefly presented static objects can be influenced by nearby moving contours, as demonstrated by various illusions collectively known as motion-induced position shifts. Here we use a stimulus that produces a particularly strong effect of motion on perceived position. We test whether several regions-of-interest (ROIs), at different stages of visual processing, encode the perceived rather than retinotopically veridical position. Specifically, we collect functional MRI data while participants experience motion-induced position shifts and use a multivariate pattern analysis approach to compare the activation patterns evoked by illusory position shifts with those evoked by matched physical shifts. We find that the illusory perceived position is represented at the earliest stages of the visual processing stream, including primary visual cortex. Surprisingly, we found no evidence of percept-based encoding of position in visual areas beyond area V3. This result suggests that while it is likely that higher-level visual areas are involved in position encoding, early visual cortex also plays an important role.

## Introduction

To perceive the world correctly, we must know not only what objects are present in a visual scene, but also where they are located. How does the visual system code location? Neurons throughout the visual system are activated by stimuli falling on specific regions of the retina, their receptive fields. It might seem reasonable that this explicit location information—the receptive field locations of cells that respond to an object—would specify an object's perceived location. However, a wide range of motion-induced position shift effects have demonstrated that motion can influence perceived location to a surprising extent (Whitney, 2002; Eagleman and Sejnowski, 2007). Indeed, a moving object may be perceived at a location quite distant from its current location in the visual field and the receptive fields that would be activated on the basis of retinal input alone.

Ramachandran and Anstis (1990) and De Valois and De Valois (1991) showed that the perceived position of a physically stationary aperture or window appears displaced in the direction of a moving texture within the window—we will refer to as “the Moving Window Effect.” In the “Flash Lag Effect,” a briefly presented, stationary stimulus is perceived as lagging behind a moving stimulus, although they are physically aligned (MacKay, 1958; Nijhawan, 1994). A slight change in stimulus configuration, however, will lead to the “Flash Drag Effect,” in which the briefly presented flash is shifted in the direction of motion of an adjacent texture, in the absence of spatial overlap between object and motion (Whitney and Cavanagh, 2000). For a more detailed discussion of these effects, we recommend the review by Eagleman and Sejnowski (2007).

**Figure 1:**
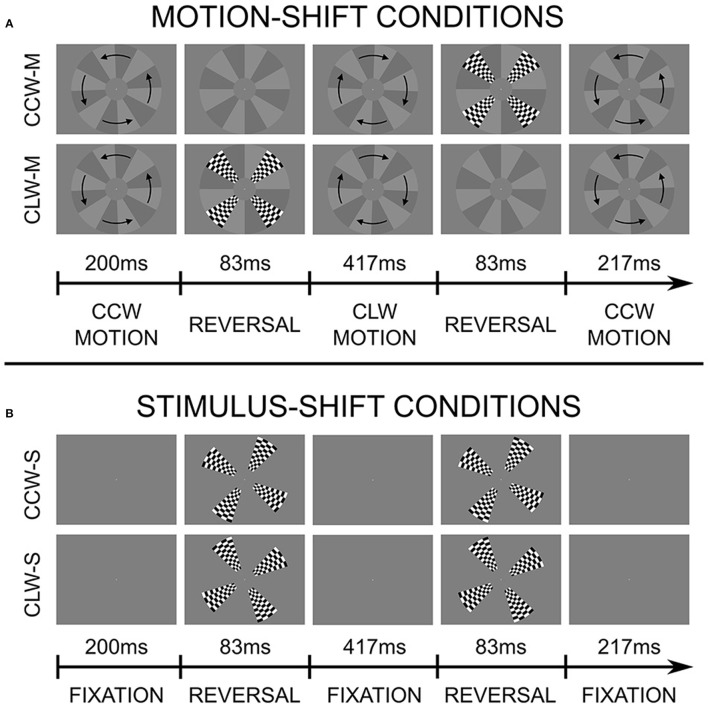
**Stimulus and experimental design**. Stimuli presented during motion-shift conditions **(A)** and stimulus shift conditions **(B)**. Each row is a separate condition, and illustrates a single 1-s cycle. Two cycles were completed for each 2-s TR. Flashes were presented once per second in motion-shift conditions, and twice per second during stimulus shift conditions. In this illustration, the offset of the checkerboard pattern in the stimulus-shift conditions corresponds to the average effect size reported by participants. The moving background always paused for 5 frames (~83 ms) at the reversal position, regardless of whether a flash was presented or not. Note that the starting direction of motion was randomly assigned for each run. The two motion-shift conditions can be seen by viewing Supplementary Videos 1, 2.

The goal of the current experiment is to use functional MRI to investigate how and where these motion-induced position shifts affect position coding. Specifically, we are interested in identifying areas in visual cortex where the illusory, motion-shifted flash positions are represented in the same way as physical positions. We base our stimulus on the “Flash Grab Effect,” a recently discovered, particularly strong example of motion-induced position shifts, where the moving stimulus undergoes a direction reversal, and a flash is presented briefly at the same time and position as the reversal. The result is that the flash appears shifted in the direction of motion following the reversal (Cavanagh and Anstis, 2013). The stimulus is shown in Figure 1 and Supplementary Videos 1 and 2, and described in more detail in the Materials and Methods Section.

Several previous studies have used fMRI to study the encoding of perceived position in the visual system. One study analyzed displacements in the perceived position of counter-phase flickering Gabor patches, relative to their actual position. They found that patterns of activity in regions of interest later in the visual processing stream such as the human homolog of motion-sensitive medial temporal cortex in macaques (hMT+), the lateral occipital cortex (LOC), and the parahippocampal place area (PPA), were most consistent with representations of the perceived, non-veridical positions, whereas patterns in early visual areas corresponded more closely to the actual physical positions of the Gabor patches (Fischer et al., 2011). Other studies have used variations of motion-induced position shifts to study perceived position: The Moving Window Effect has been used to identify a paradoxical effect in early visual cortex, where patches containing motion away from fixation, were perceived to be shifted away from fixation, but elicited more central activation in retinotopic cortex, while patches containing motion toward fixation, that were perceived as shifted in that direction, led to more peripheral activation (Whitney et al., 2003). Related effects have been reported in multiple studies over the years (Liu et al., 2006; Wang et al., 2014; Harvey and Dumoulin, 2016), although the interpretation of these effects and their relation to representations of object position in early visual areas is still disputed. Finally, a recent study used the Flash Drag Effect to show that activity in visual areas hMT+ and V3A was correlated with perceived position, whereas activity in early visual cortex was not (Maus et al., 2013a).

Transcranial magnetic stimulation (TMS) has also been used to directly investigate the contribution of different visual cortical areas to various types of motion-induced position shifts. McGraw et al. (2004) investigated a phenomenon where the perceived position of a static feature was shifted following motion adaptation (Snowden, 1998; Nishida and Johnston, 1999; McGraw et al., 2002) and found that TMS to hMT+ led to a significant reduction in the adaptation-induced position shift, whereas TMS to primary visual cortex (V1) had little effect (McGraw et al., 2004). These findings were replicated in another study, which also found that TMS to hMT+ abolished biases in reaching movements following motion adaptation (Whitney et al., 2007). More recently, Maus and colleagues applied TMS to participants while they made judgments about the position of a moving stimulus in a version of the Flash Lag Effect. They found that applying TMS to hMT+ reduced the effect, but that applying TMS to early visual areas did not (Maus et al., 2013b). Although it is not clear from these studies whether motion processing or position encoding was disrupted by TMS, they do suggest that hMT+ plays an important role in motion-induced position shifts.

In summary, a range of evidence suggests that perceived position is represented in areas late in the visual processing stream, with hMT+ as perhaps the strongest candidate area for representing motion-induced position shifts. What could be gained by probing position encoding using the Flash Grab Effect? The Flash Grab Effect is an order of magnitude larger than the Flash Drag Effect (Cavanagh and Anstis, 2013), and larger than any of the effects that have previously been studied using TMS and fMRI, which means that an experiment using the Flash Grab Effect has the potential to detect fMRI effects that other studies may have missed.

Our experimental design and analysis followed the strategy used by Maus et al. (2013a) closely, except that we used the Flash Grab Effect instead of the Flash Drag Effect. In addition to the two motion-induced position shift conditions, we included two conditions in which the checkerboard was physically shifted in either the clockwise or counter-clockwise direction (CLW-S and CCW-S, respectively), as well as conditions in which the motion stimulus (M) and the fixation spot (F) were presented in isolation. This design makes it possible to correlate the multivariate patterns of activity evoked by motion-induced position shifts, with patterns evoked by corresponding physical shifts in position. If an area encodes perceived, rather than physical position, one would expect the activation patterns generated by motion-induced shifts to be strongly correlated with those generated by physical shifts. Performing this analysis within several functionally defined visual regions-of-interest (ROIs) allowed us to identify such areas, while avoiding artifacts from the motion stimulus as well as other potentially confounding factors.

## Materials and methods

### Participants

Seven participants (ages 24–31, mean age = 28, one female) took part in the experiment. All were members of the Dartmouth College community with normal or corrected to normal vision that volunteered to participate. Each participant gave written informed consent prior to the experiment and was compensated with $20 per hour.

### Stimulus presentation

Stimuli were projected onto a screen behind the MRI scanner bore which participants viewed through a mirror mounted on the head coil. The screen resolution was 1,600 × 1,200 pixels and the screen was running with a 60 Hz refresh rate. Stimuli were generated and presented using the Psychophysics Toolbox, version 3 (Brainard, 1997; Pelli, 1997), on a PC running MATLAB R2010a (MathWorks, Natick, MA) in Ubuntu Linux.

### Stimulus

The stimulus was based on the Flash Grab Effect discovered by Cavanagh and Anstis (2013). The moving stimulus was a disc with 12 wedge-shaped sectors alternating between light and dark gray, rotating on a gray background (see Figure 1 and Supplementary Videos 1, 2). The disc covered the entire vertical span of the screen's field-of-view (radius: 8.5°/visual angle), except for a central region (radius: 2.1°/visual angle), which had the same color as the background (see Figure 1). The disc was presented at the center of the screen, and oscillated back and forth at 2.30° of rotation per frame, traveling 60° of rotation between each direction reversal, and completing two reversals per second. When the disc reached the point of reversal, it remained there, stationary for 5 frames (~83 ms), while a checkerboard pattern was presented such that it completely filled in four sectors, centered on 45, 135, 225, and 315°.

When the checkerboard pattern was presented at the end of a counter-clockwise rotation, its perceived position was shifted in the clockwise direction, and when it was presented at the end of a clockwise rotation, the shift was perceived in the counter-clockwise direction. These were our two main experimental conditions. Importantly, the checkerboard stimulus can be shifted in different directions (clockwise or counterclockwise), inducing a large difference in perceived position, by simply changing the presentation timing, while the oscillatory motion of the background, and everything else, is kept constant.

### fMRI acquisition parameters

Functional Magnetic Resonance Imaging scans were acquired on a Philips Achieva 3.0 Tesla scanner (Philips Medical Systems, Bothell, WA), with a 32-channel SENSE birdcage head coil. Structural T1-weighted images were collected using a 1 × 1 × 1 mm voxel resolution 3D magnetization-prepared rapid gradient echo sequence, with standard parameters. Functional images were acquired with a T2^*^-weighted echo planar imaging sequence (repetition time = 2 s, echo time = 35 ms, flip angle = 90°). We collected 35 interleaved, horizontally oriented slices (in plane resolution, 3 × 3 mm; slice thickness, 3 mm; no gap between slices, in-plane FOV = 240 × 240, in-plane matrix 80 × 80) that covered all of the occipital and most of the parietal cortex but were missing inferior parts of the temporal and frontal cortices. For each participant, we collected 10 runs, acquiring 184 TRs in each.

### Psychophysics procedure and analysis

Prior to the fMRI experiment, we conducted a simple psychophysical experiment inside the scanner to measure the extent to which our stimulus elicited the Flash Drag Effect in each of our participants. On each trial, participants viewed the stimulus in either the clockwise or counter-clockwise condition for as long as they wanted while maintaining central fixation. They were asked to rotate the entire stimulus (offsetting both the checkerboards and the reversal positions of the rotating background) using the left and right arrow keys until the checkerboard pattern appeared un-tilted. The stimulus was always rotated by a random amount between 0 and 20° either to the left or the right at the beginning of each trial, to avoid any influence of participant biases. When participants were satisfied with their adjustment, they advanced the trial by pressing the enter key. Participants completed three adjustments for each condition, which were averaged to get the effect size for each participant.

### fMRI procedure

For the fMRI experiment we used six different stimulation conditions, presented in randomly interleaved 12 s blocks: clockwise motion-induced position shift, counter-clockwise motion-induced position shift, clockwise physical shift, counter-clockwise physical shift, motion alone and fixation alone. In all three motion conditions, the disc always started at the same rotation angle, and moved 45° before reversing for the first time, always completing two reversals per second. The starting direction of motion was randomly assigned for each run. This meant that during both motion-induced shift conditions, the checkerboards' sectors were always presented exactly once per second and were always on for 5 frames (~83 ms) per presentation. Checkerboards always occupied the same physical position, regardless of the perceived shift direction. In the motion-alone condition, the disc moved in the exact same way, but the checkerboard pattern was not presented. In the physical shift conditions, the checkerboards were presented without motion, and physically rotated in the clockwise or counter-clockwise direction. The size of the physical shift was adjusted to correspond to the effect sizes measured psychophysically for each individual participant. The adjustment was done independently for each shift direction, so any asymmetry in effect size between the clockwise and counter-clockwise motion-induced position shift would lead to a corresponding asymmetry in the physical shift. In the physical shift conditions checkerboards were presented twice as often as in the motion-induced shift conditions, at 2 Hz. This was done so that a smaller number of block repetitions could be used for these conditions, compared to the motion-induced shift conditions, without a concomitant drop in signal strength. In the fixation condition, the fixation spot was presented alone. Each of the stimulus conditions was shown continuously for an entire 12-s block, and there was no gap between blocks. The motion-induced position shift conditions were repeated seven times, whereas all other conditions were repeated four times. Each run started and ended with 2 TRs of fixation alone, and the total run length was 184 TRs. Each participant completed a scanning session of 10 runs, and performed a relatively demanding task at the central fixation cross throughout each run. The light gray fixation cross (0.23 × 0.23°/visual angle) increased or decreased in contrast randomly every 4–8 s (at least once during every stimulation block). Participants had to indicate these contrast changes by pressing two separate buttons on a response box for increases and decreases in contrast, respectively.

### Region-of-interest definition

All analyses were performed within bilateral ROIs, which were defined based on data from separate scanning sessions. Visual cortical ROIs V1, V2, and V3 were defined using retinotopic mapping methodology, as described previously (Sereno et al., 1995; Slotnick and Yantis, 2003; Caplovitz and Tse, 2010). We used 22.5° rotating monochromatic checkerboard bowties and rings. Both occupied 16 non-overlapping positions on each cycle, remained at each position for a single 2-s TR, and completed five 32-s cycles per run. Bowties extended from the center of the screen all the way to the edge of the field of view (total field of view size: 22.7 × 17° of visual angle), and traveled clockwise and counter-clockwise on alternating runs, while rings expanded and contracted. To ensure that participants were awake and fixating during retinotopic mapping, participants reported brief fixation color changes with a button box throughout the runs. Cortical reconstruction and volumetric segmentation, as well as cortical inflation and flattening, were performed using the FreeSurfer image analysis suite (Dale et al., 1999; Fischl et al., 1999). Once flattened cortical surface models of the occipital lobes had been created using FreeSurfer, all surface models were imported into SUMA (Saad et al., 2004). Retinotopic time series data were analyzed using AFNI's @Retinoproc program. This program preprocessed the data, mapped data onto Surfaces, smoothed the data along the surface (6 mm FWHM) and computed average phase maps for wedges and rings, as well as visual field sign maps, as described by Warnking and colleagues (Warnking, 2002). ROIs were drawn by hand based on the phase maps and the visual field sign maps, and then mapped back into the volume space.

The hMT+ ROI was defined based on a localizer stimulus consisting of black and white random dots on a gray background that were either stationary or oscillated between expanding and contracting motion on alternate 2-s TRs (speed of motion, 2.56°/visual angle per second), with the position of all dots being reassigned every five frames. Static and moving dots were shown for 20 s blocks, with 16-s gaps between them. Throughout each run, participants performed a fixation task identical to the one used during retinotopic mapping. To define hMT+, we preprocessed the EPI scans and performed a GLM contrast to identify voxels that had a bigger response to moving dots than static dots.

In addition to the functionally defined ROIs, we also used an anatomically defined ROI, which covered the intraparietal and transverse parietal sulci (label = “S_intrapariet_and_P_trans;” Destrieux et al., 2010) defined using an automatic parcellation technique implemented in FreeSurfer (Fischl, 2004; Destrieux et al., 2010). We were specifically interested in the intraparietal sulcus (IPS) because this area is positioned relatively late in the dorsal visual processing stream, and has a topographical map structure similar to that seen in occipital cortex (Swisher et al., 2007), two features that in combination make IPS a likely candidate for coding perceived position.

Finally, to exhaustively test the set of topographically organized areas in visual cortex, we also derived a larger set of visual ROIs from a probabilistic atlas generated by Wang et al. (2015). They used retinotopic mapping to define 25 topographic ROIs covering 22 visual areas in ~50 individual participants, converting the surface data from each individual to surface-based standardized space (Argall et al., 2006), and then assessing the likelihood, across participants, of any particular vector on the standardized surface belonging to a particular ROI (Wang et al., 2015). The atlas was defined using a maximum probability approach, which considers a given vector as part of the set of ROIs if it is more often found within the set, than outside the set, across participants. If this is the case, the vector is then assigned the value of the most likely ROI, and if not, it is considered to be outside the set of ROIs. The maximum probability approach captures much of the overall structure of ROIs defined for individual subjects and generalizes well to novel participants that did not contribute to the atlas generation (Wang et al., 2015). We downloaded the atlas from http://scholar.princeton.edu/sites/default/files/napl/files/probatlas_v4.zip and converted the ROIs from standardized surface space to native surface space for each of our participants, using nearest-neighbor interpolation. We used surface-based clustering to eliminate vertices more than one edge removed from the main cluster of each ROI, to ensure that all ROIs consisted exclusively of contiguous vertices. This step eliminated small isolated speckles, while having minimal effect on the overall structure and extent of the ROIs. Finally, we converted the ROIs from surface-space to volume space, and aligned them to the experimental data.

### fMRI analysis

Most analysis steps, including preprocessing, alignment, and general linear model computation were done using the afniproc.py framework, which utilizes multiple different programs from the AFNI software package (Cox, 1996). Pre-processing steps included motion registration of all TRs in all EPI scans to the third TR of the first EPI scan, and scaling the EPI mean of each voxel to 100. Slice-timing correction was done automatically as data was exported off the scanner computer. No smoothing, resampling or spatial normalization of the data was performed.

High-resolution T1-weighted structural scans collected at the end of each experiment scanning session were aligned to the EPI scans. This was done to aid alignment between EPI scans and functional ROIs defined based on data from separate scanning sessions. After the structural scan collected during the experiment had been aligned to EPI scans, a structural scan associated with the ROIs was aligned to the experiment structural scan, using a six parameter rigid-body transformation implemented in AFNI's align_epi_anat.py (Saad et al., 2009). Then, the same transformation was applied to the ROIs so that they were aligned to the EPI scans. After alignment, all ROIs were resampled to match the resolution and extent of the experiment data, and any overlapping voxels between adjacent ROIs within a set were removed to guarantee a conservative specification of each ROI.

Data from all EPI scans were then entered into a General Linear Model, which included six regressors of interest (CCW-M, CLW-M, CCW-S, CLW-S, motion alone, and fixation), as well as regressors of non-interest, which consisted of four run-wise baseline parameters corresponding to constant signal, linear drift, and 2nd and 3rd degree polynomials for each run, as well as six motion registration parameters across all runs. To further eliminate any influence of head-motion-related signal on the analysis, the regression identified motion-outlier TRs by taking the Euclidean norm of the derivative of the six motion parameters, while ignoring shifts between runs. If the Euclidean norm exceeded 0.3 (the AFNI default limit) for a given TR, that TR, as well as the TR before, was excluded from the regression. The mean proportion of TRs censored across all runs was 11.5%. The GLM design matrix was created using AFNI's 3dDeconvolve program, and then passed to 3dREMLfit, an AFNI program that does a GLM using ordinary least squares regression, and accounts for serial auto-correlation in the data. Beta coefficients for each of the six conditions were computed for each voxel across all of our visual ROIs, and were then loaded into MATLAB (The MathWorks; Natick, MA) where all of the following analyses were performed.

Prior to any further analysis, we performed a voxel selection step within each of our ROIs. Only voxels in which the contrast (CLW-S+CCW-S) > fixation was significant (Bonferroni corrected *p* < 0.01), that is, voxels that had a larger response to either of the two physical shift conditions than to fixation alone, were included in the rest of the analysis. This procedure identified voxels that responded to the physical locations in the visual field that we were interested in, and excluded voxels in regions of cortex that had little or no response to the flashed checkerboards. The average proportion of ROI voxels included by our voxel selection step across participants was fairly similar across our four main ROIs (V1: 44.1%, V2: 42.1%, V3: 48.8%, hMT+: 49.6%), but in anatomically defined IPS the proportion was much smaller (18.9%). For the atlas-defined ROIs, to ensure that enough voxels would survive the voxel selection step, we considered the six IPS-regions as a single region, and also combined areas TO1 and TO2. This led to the following average proportions across atlas-defined ROIs: V1: 40.5%; V2: 45.9%; V3: 54.2%; V3A: 50.1%; V3B: 55.5%; V4: 58.6%; VO1: 67.5%; VO2: 45.3% LO1: 63.9%; LO2: 44.6%; TO: 46.0%; IPS0-5: 29.3%. All of the following ROI analyses were done within voxels selected by this procedure, unless stated otherwise.

In order to isolate the signal from the flashes, we then computed differences in GLM beta coefficients for each voxel between the CLW-M and CCW-M conditions. Because the physical position of the flashes and the background motion across a block were identical in the two conditions, the resulting difference maps defined by the CLW-M—CCW-M GLM contrast exclusively reflect the effect of the motion-induced shift on the position of the flashes, whether clockwise or counterclockwise. To similarly assess the effect of the physical shift in stimulus position, we subtracted the GLM beta coefficients for CLW-S and CCW-S, producing a difference map that reflected the difference in flash representations between stimulus shift conditions. We evaluated the similarity between difference maps evoked by illusory, motion-induced position shifts and difference maps evoked by shifts in physical stimulus position, by computing the correlation between CLW-M and CCW-M difference maps and CLW-S—CCW-S difference maps within each of our ROIs. Because the physical stimulus positions were identical in CLW-M and CCW-M, an area that codes physical position exclusively should produce difference map correlations that are no different from chance. If an area does code perceived position, however, the difference maps evoked by illusory perceived position shifts should be similar to those evoked by physical position shifts, and correlations should be positive. Following all correlation analyses, Pearson's correlation coefficients (*r*-values) were converted to Fisher z′ scores to allow linear comparisons of values.

### Significance assessment

We assessed the significance of the difference map correlations by first performing permutation testing in the following manner. For each participant, we randomly shuffled the condition labels for our CCW-M and CLW-M conditions, while keeping the labels for the four other conditions intact. This procedure eliminates any information about the motion-induced position shift that potentially exists in the data, without compromising any of the other conditions. We then performed the GLM in the exact same way as for the original data, with the same regressors of non-interest and motion censoring. We did this 1,000 times and extracted beta coefficients from the voxels that survived voxel selection in each of our functionally defined ROIs and correlated the difference maps for each permutation to obtain a distribution of correlations for each participant. Because we shuffled the labels in the shift conditions, this distribution of z′ scores now represents the null hypothesis that the difference maps for the physical shifts and motion-induced shifts are not correlated, without making any assumptions about the underlying distribution. Thus, the proportion of these samples that led to higher z′ scores than those obtained with veridical, unshuffled labels represents the likelihood of incorrectly rejecting the null hypothesis.

We used two approaches to assess the group-level statistical significance of our findings. The first was a bootstrap approach identical to the one taken by Maus et al. (2013a). We randomly selected one sample from the distribution of shuffled z′ scores, subtracted these values from the unshuffled z′ score, did this for all seven participants, and calculated the mean difference across participants. We then repeated this measure 1,000 times. If this distribution is bigger than zero, it means that the measured, unshuffled z′ scores are higher than would be expected under the null hypothesis, and the portion of this distribution that is smaller than zero, can be considered a *p*-value for the hypothesis that the z′ score is larger than would be expected due to chance. One potential danger of this approach is that it effectively disregards individual participant differences, by calculating a distribution of mean differences across participants. This means that if one or two participants have much higher correlation scores than the rest, they can shift the whole distribution of mean differences away from zero and potentially lead to inappropriately small *p*-values. To ensure that this was not the case with our data, we performed an additional test for significance in the following way: First, we calculated the mean of the distribution of shuffled samples for each participant. We then performed a paired *t*-test across our participants between the measured, unshuffled correlation and the shuffled mean correlation. The bootstrap and paired *t*-tests approach should be complementary, and if a real effect exists within an ROI, we would expect both to be significant.

### ROI control analysis

In order to test whether or not the observed correlations were specific to our selected ROIs, we performed the same correlation analysis within randomly selected clusters of contiguous voxels within a gray matter mask, defined for each participant based on a gray matter segmentation process performed in FreeSurfer, and resampled to match the voxel size used in our experiment. In order to allow comparisons across participants, we spatially normalized (Talairach and Tournoux, 1988) experiment structural scans for each participant, using an average of 152 normal participant brains, matched to MNI standard space (Evans et al., 1993) as the template. We then applied the same transformation to the gray matter mask and the statistical data. To get a shared gray matter mask across all participants, we created a new mask in which a voxel was only included if it was present in at least four individual participant masks. We now randomly selected a seed voxel within this cross-participant gray matter mask and defined an ROI by growing a sphere around the seed voxel until the number of gray matter voxels corresponded to the average number of selected voxels within V1 (116 voxels). We did this 1,500 times, and computed the average correlation across participants within each random gray matter ROI. To rule out any advantage of our ROIs over the rest of the brain due to voxel-selection, we also did a separate test in which the number of voxels in randomly selected ROIs were made to correspond to the average number of voxels in the original V1 ROI (264 voxels), and voxel selection was performed within each random ROI by the same criterion as was used in our main analysis, prior to computing the correlation. Some clusters would have two or fewer voxels that survived the selection criterion, for some participants, which meant that those clusters had to be excluded from the analysis. To avoid having a few participants skew the distribution of correlations, we only included averages from clusters that survived voxel selection in four or more participants. Using this procedure, 467 of the 1,500 clusters survived. For both these analyses, the distribution of correlations formed a null distribution against which we could test whether the correlation effect was specific to visual cortex ROIs.

## Results

### Psychophysical results

**Figure 2:**
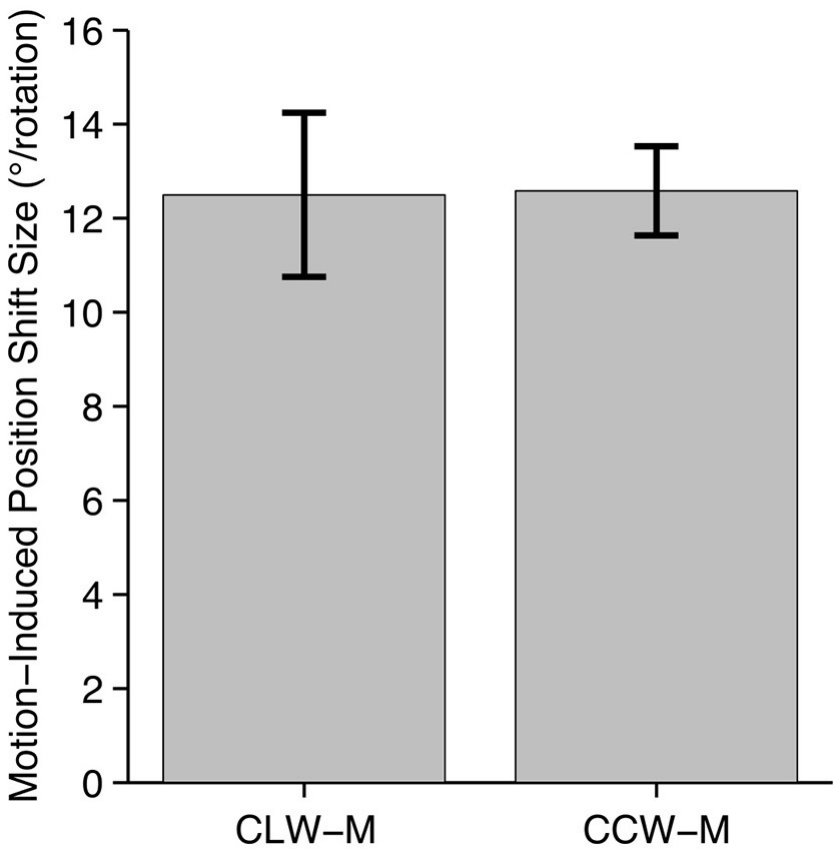
**Psychophysical data**. Average size of the motion-induced position shift across seven participants, based on data collected inside the scanner, prior to the fMRI experiment. Large, almost perfectly equivalent shifts were seen in both directions. Although the two conditions produced shifts in opposite directions, they are presented here as absolute values, to aid comparison.

Average results of the psychophysical experiment are plotted in Figure 2. There was substantial variability among participants, with mean effect sizes ranging from 3.92° to 17.4° for CLW-M and 8.45° to 17.0° for CCW-M. The two shift directions produced similar effect sizes averaged across participants (CLW: 12.5°; CCW: 12.6°) and there was no significant difference in effect size between the two directions [*t*_(6)_ = 0.0555, *p* = 0.958]. Participants performed the psychophysical experiment prior to scanning, and the physical shift in conditions CLW-S and CCW-S during the fMRI experiment was matched to the perceptual effect size of each individual participant.

### Multivariate pattern analysis

**Figure 3:**
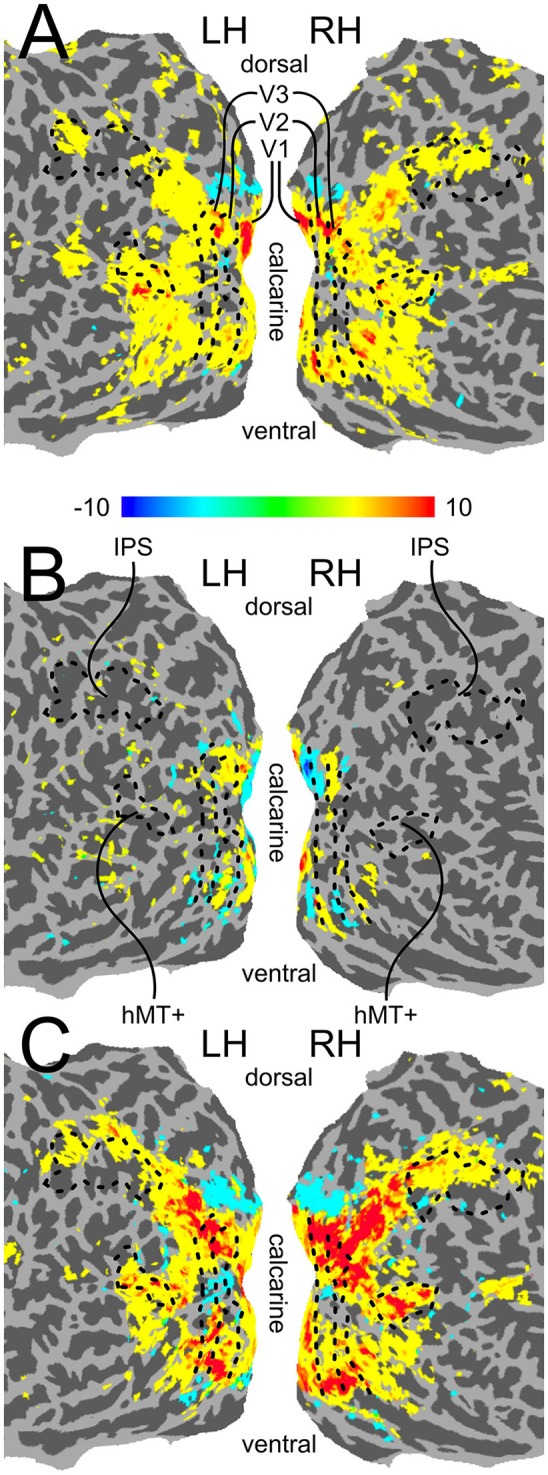
**Flattened surface activation maps. (A)** The contrast (CLW-S+CCW-S) > Fixation, which served as the voxel selection criterion for our multivariate pattern analysis. **(B)** The contrast CLW-S > CCW-S, indicating differential activity evoked by the two physical shift directions. **(C)** The contrast Motion > Fixation. All three maps were created by mapping GLM contrasts from volume space, thresholded at Bonferroni corrected *p* < 0.01, onto flattened surfaces generated in FreeSurfer, using AFNIs 3dVol2Surf program. The boundaries between retinotopic regions V1–V3, as well as the location of functionally localized hMT+ and the anatomically localized IPS ROI, are indicated with a black outline.

As mentioned above, we performed our main analysis within five independently defined ROIs: V1, V2, V3 (defined with standard retinotopic mapping), a motion-sensitive area in medial temporal cortex (hMT+; defined using a functional localizer), and an anatomically defined area in the intra-parietal sulcus (IPS; see Section Region-of-Interest Definition for details on how all ROIs were defined). The boundaries of the five ROIs are shown on the flattened cortical surface of an example participant in Figure 3. The contrast used for our voxel selection step (see Section fMRI Analysis), as well as contrasts indicating differential activity evoked by the two physical shift directions and activity evoked by the motion stimulus, is also shown in Figure 3. The checkerboard wedges were centered in each quadrant of the visual field, and, as expected, led to activity near the centers of the quarter-field representations in areas V1-V3 (see Figure 3A). Differential responses to the two physical shift conditions were mostly seen in early visual cortex (see Figure 3B), while the motion condition led to more widespread activity across visual cortex (see Figure 3C).

**Figure 4:**
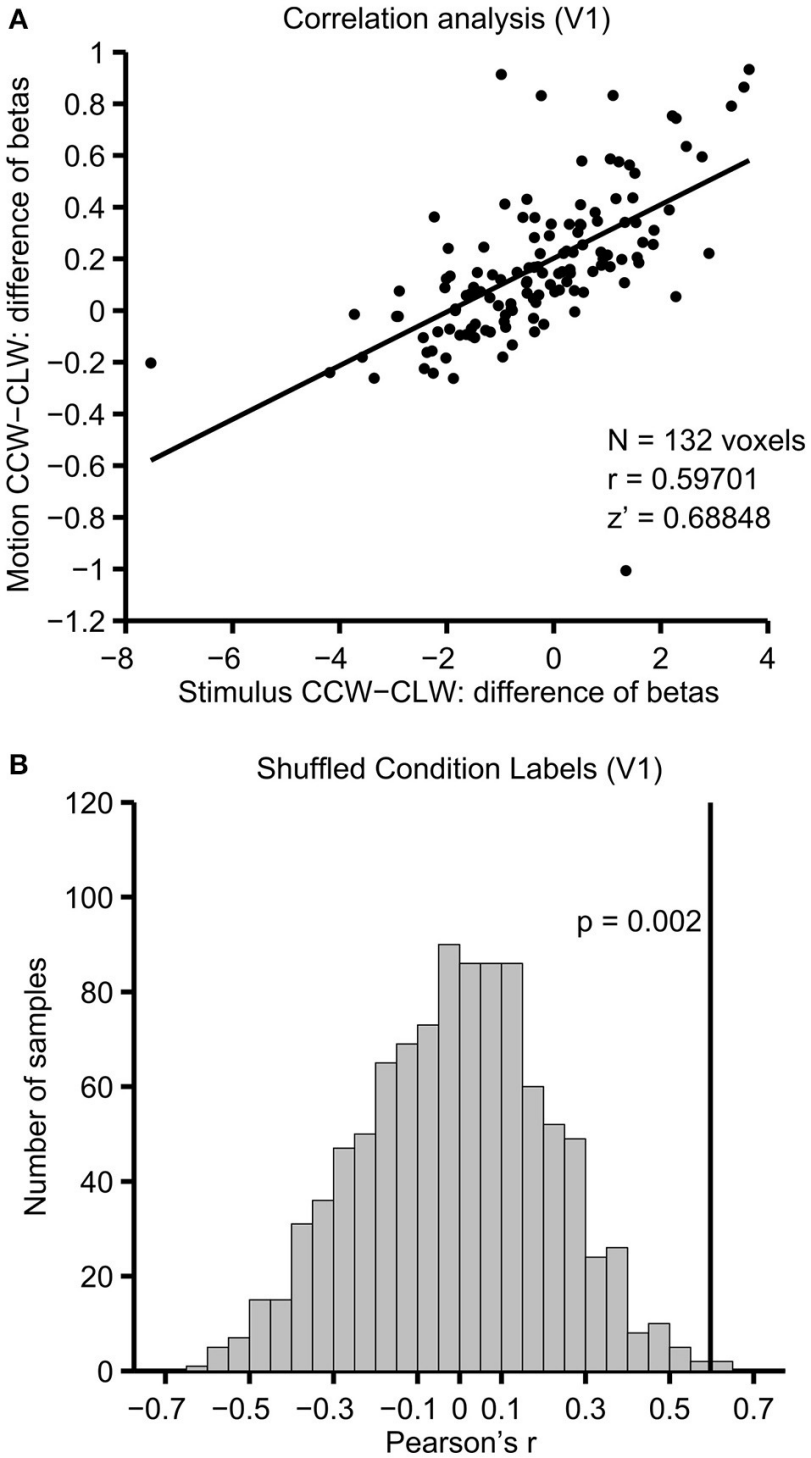
**Correlation and permutation testing**. Illustrated for V1 in a single example participant. **(A)** The difference of beta coefficients between motion conditions (y-axis) and physical stimulus shift conditions (x-axis) for each of the 132 voxels that survived the voxel selection procedure in V1 of this participant. **(B)** Correlations resulting from repeating this analysis 1,000 times, while shuffling the labels between CLW-M and CCW-M. The unshuffled correlation, illustrated with a solid line, was higher than all but 2 of the shuffled correlations, indicating that the correlation is stronger than what would be expected given the null hypothesis that there is no information shared between the motion and stimulus shift contrasts.

The analysis steps taken for each individual participant (see Section fMRI Analysis) are illustrated in Figure 4, using data from the V1 ROI of an example participant. The difference of beta coefficients for physical stimulus shift conditions is plotted against the analogous difference for motion-induced position shift conditions in Figure 4A, with each point representing one voxel. The Pearson's *r* for the correlation was 0.597, which corresponds to a Fisher's z′ of 0.688. The null distribution of correlations produced by shuffling the labels of the motion-induced position shift conditions is seen in Figure 4B. In V1 of this participant, the unshuffled correlation (indicated with a solid black line) clearly lies at the extreme positive end of the distribution of shuffled correlations, and is thus larger than one would expect given the null hypothesis that the ROI has no information about motion-induced position shifts.

**Figure 5:**
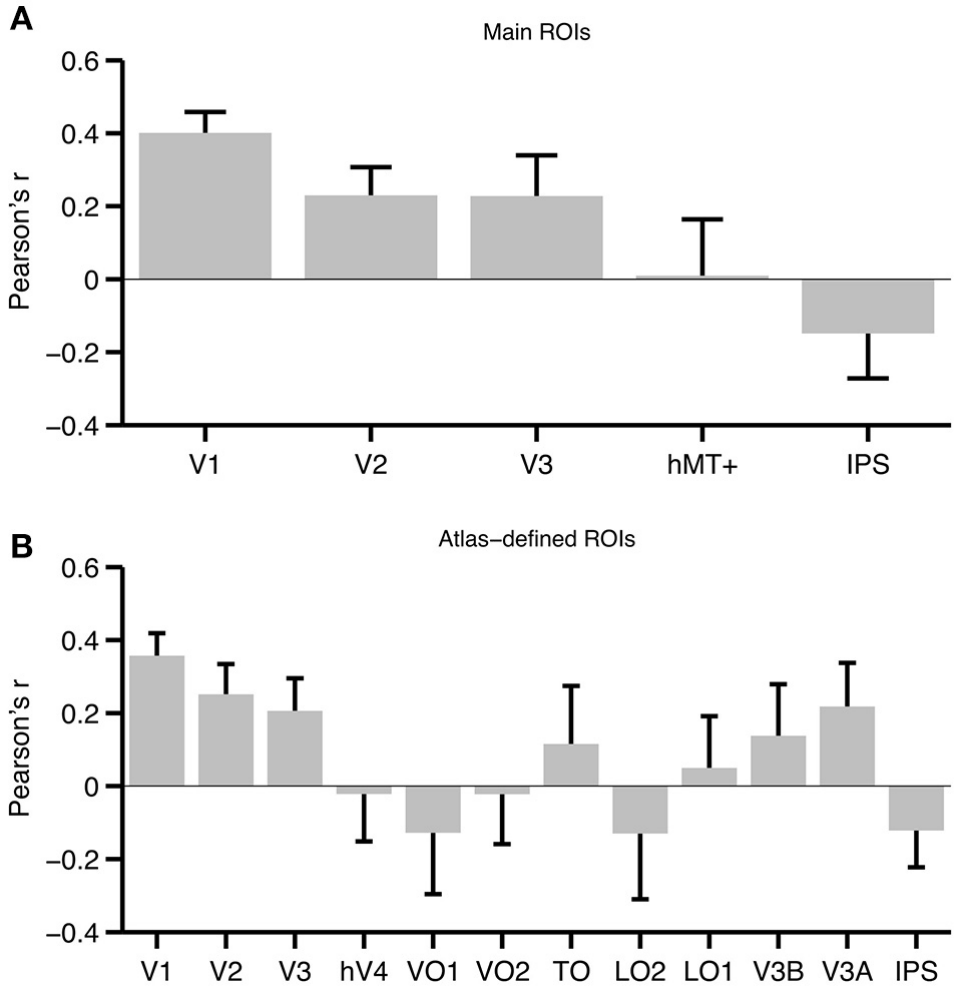
**Average correlation across participants for each ROI. (A)** Average correlations for the main set of ROIs. **(B)** Average correlations for ROIs defined based on a probabilistic atlas (Wang et al., 2015). Note that the atlas-defined TO ROI is the union of TO-1 and TO-2, while IPS is the union of IPS0-5. Error bars represent the standard error of the mean. To aid interpretation, correlations are plotted as Pearson's *r*, but we emphasize that these values were converted to Fisher's z′ prior to statistical analysis.

V1 had the highest average correlation across participants (see Figure 5A). We assessed group level significance using a bootstrap approach as well as a paired *t*-test (each is described in more detail in the Materials and Methods Section). The correlation in V1 was highly significant, both when using the bootstrap approach (*p* < 0.001), and when using a paired *t*-test [*t*_(6)_ = 6.30, *p* = 0.0004]. Both tests were also significant in V2 [bootstrap: *p* = 0.001, paired *t*-test: *t*_(6)_ = 3.00, *p* = 0.0119] and V3 [bootstrap: *p* < 0.001, paired *t*-test: *t*_(6)_ = 2.00, *p* = 0.0443]. Importantly, hMT+ or IPS did not reach significance using either the bootstrap (hMT+: *p* = 0.455, IPS: *p* = 0.932) or paired *t*-test approach [hMT+: *t*_(6)_ = 0.249, *p* = 0.406; IPS: *t*_(6)_ = −1.29, *p* = 0.877].

To exhaustively test the set of topographically organized areas in visual cortex, we also analyzed 12 ROIs defined using a probabilistic atlas. The results from this larger ROI set mirrored our findings from the main set (see Figure 5B). Atlas-defined V1, V2, and V3 all showed significant effects both when using the bootstrapped approach and when using a paired *t*-test. There was also a marginally significant effect in V3A [bootstrap: *p* = 0.026, paired *t*-test: *t*_(6)_ = 1.80, *p* = 0.061], but all other ROIs were far from significance (all *p*'s > 0.15).

### Estimating effect size

To estimate the size of the position shift effect in stimulus units, we first computed the mean absolute deviation from zero of the difference maps for each participant, separately for the physical and perceptual shift conditions. This provides a participant-specific estimate of the fMRI effect size for the perceptual and physical shifts. We focused on functionally defined V1, the area where we saw the biggest effect in the correlation analysis. The effect size for the perceptual shift was 19.8% (SE = 2.9) of the effect size for the physical shift, corresponding to a shift of 4.67° (SE = 0.59) of rotation. This result demonstrates that despite the high correlations we saw in V1 and other early visual areas, representations of motion-induced shifts in these areas are not necessarily completely analogous to representations of the corresponding physical shifts.

### Performance on scanner task

Participants generally performed well on the contrast change detection task in the scanner; the percentage of correct responses across runs was never below 75% correct for any of our participants. To test for differences in fixation spot performance between experimental conditions we performed a two-way repeated measures ANOVA on the run-wise mean accuracies across participants, with task type (contrast increments vs. decrements) as the first level, and condition as the second level. We found a main effect of contrast task type [*F*_(1, 30)_ = 6.775, *p* = 0.0405], indicating that participants were worse at detecting contrast increments compared to decrements, but no other significant main effects or interactions. Importantly, this indicates that there were no systematic differences in attention between our conditions. We explicitly tested the possibility that attention was somehow influencing our correlation analysis by comparing the average performance across task type between CLW-M and CCW-M conditions, and separately between CLW-S and CCW-S conditions. If both of these tests are significant, attentional differences could drive the correlations. In fact, both tests were non-significant [S: *t*_(6)_ = 1.038, *p* = 0.339; M: *t*_(6)_ = 1.055, *p* = 0.332]. We thus conclude that it is unlikely that our correlations were influenced by attention.

### Control analyses

We did additional analyses to determine whether differences in overall signal-to-noise ratio (SNR) or number of voxels between the ROIs could drive the correlation differences we found. We calculated SNR as *t*-values for the contrast of all stimulus conditions (CLW-M, CCW-M, CLW-S, CCW-S, and M) > Fixation, and correlated the average SNR within each ROI, for each participant, with the unshuffled correlation scores. We determined significance using a similar permutation analysis to the one used for our main effect. The SNR values were shuffled among ROIs for each participant 1,000 times, and *p*-values were computed as the ratio of shuffled correlations that were lower than the unshuffled correlations. Significance was determined using the same approach for all control analyses. The correlation with SNR was not significant (*r* = −0.074, *p* = 0.67), indicating that the differences cannot be explained by differences in overall signal quality.

Our results could not be explained by number of selected voxels or overall ROI size. We correlated the number of selected voxels within each ROI with the contrast correlation scores across our five main ROIs and all participants. There was no significant correlation, indicating that number of voxels did not predict the size of the correlation (Pearson's *r* = −0.021, *p* = 0.565). We did the same correlation analysis using the overall ROI size prior to voxel selection, and again found no significant correlation (Pearson's *r* = −0.114, *p* = 0.823). Running the same correlations on a set of atlas-defined ROIs did not change any of the results.

We also assessed whether the differences between ROIs could be explained by variability in how well the physical stimulus shift was coded by each ROI. We used the absolute value of the *t*-score associated with the contrast CLW-S > CCW-S as an unsigned measure of each voxel's ability to code the physical shift, and averaged within each ROI. This measure was significantly correlated with the unshuffled correlations scores (*r* = 0.397, *p* = 0.006), indicating that the weaker effects we see in hMT+ and IPS may be due to a weaker retinotopic representation of the physical shift in those areas. The correlation was smaller for the full set of atlas-defined ROIs, however, and did not quite reach significance (*r* = 0.165, *p* = 0.0580), suggesting that differences in retinotopic representations may not fully account for the effect.

We tested whether the observed correlations were specific to selected set of visual ROIs, by performing the same correlation analysis within 1,500 randomly selected clusters of contiguous gray matter voxels, excluding the ROIs used in the main analysis (see Section ROI Control Analysis). We did two versions of this analysis; one where we matched the cluster size to the average number of selected voxels within V1 (116), and one where we matched cluster size to the average total size of V1, and then performed voxel selection as in the main analysis. In both analyses, correlations in random ROIs extremely rarely exceeded the average correlation found in V1 (no voxel selection: *p* < 0.001; voxel selection: *p* = 0.00214), indicating that the high correlations found in early visual cortex had clear spatial specificity for early visual cortex ROIs, and were unlikely to occur elsewhere in the brain.

To ensure that our results were not inappropriately biased by our voxel selection criterion, we split the data into odd and even runs, and computed two additional GLMs for each individual participant, based on the two split halves. We then did voxel selection on data from odd runs and computed the correlations based on data from even runs, and vice versa. To compensate for the lower number of runs, we set our voxel selection criterion at a slightly lower *t*-value (corresponding to a Bonferroni corrected *p* < 0.05) than that used in the original analysis (corrected *p* < 0.01), but otherwise the analysis was done in the same way. The average overlap between voxels selected based on independent data, and voxels selected based on the complete data set, across all ROIs was 81.5%. We performed paired *t*-tests for each ROI comparing the correlation scores based on the complete dataset with the correlation scores from odd and even runs, with voxel selection based on independent data. None of these tests were significant (smallest *p* = 0.1).

To further control for any undue influence of our voxel selection criterion, we also computed contrast correlations based on all of the voxels within our ROIs, without any voxel selection. This reduced the mean correlations in the early visual areas, but there was still a significant effect in V1 (Fisher z′ = 0.195), both when using the bootstrap approach (*p* < 0.001) and when doing a paired *t*-test [*t*_(6)_ = 4.227, *p* = 0.003]. There was also a significant effect in V2 (Fisher z′ = 0.152), but only when using the bootstrap approach (*p* = 0.05), while the V3 effect was no longer significant under this analysis. Importantly, correlations in both hMT+ and IPS remained non-significant in this analysis, indicating that neither area was negatively biased by the voxel selection procedure. This is particularly important for IPS, since a greater proportion of voxels were excluded by the voxel selection procedure for this ROI than was the case for the other ROIs. It is not surprising that including all voxels from within the ROIs would reduce the effect in early visual areas, because voxels that have little or no consistent response to the conditions of interest would be expected to add additional noise to the data going into the correlation analysis. However, the fact that selecting our voxels on independent runs does not change our pattern of results, and that our basic effect, at least in V1, persists even when all voxels are included, shows that our voxel selection criterion did not unduly bias our results.

## Discussion

The results of our correlation analysis indicate that in V1, V2, and V3, illusory, motion-induced shifts in *perceived* location produced patterns of BOLD signal similar to the patterns produced by corresponding shifts in *physical* location. This effect was exclusive to the early visual areas—no significant correlations were found in hMT+ or IPS. We also ran our correlations in a set of atlas-defined ROIs, which produced the same effects in early visual cortex as well as a marginally significant effect in V3A, but no effects in any other retinotopic area. Moreover, a permutation analysis determined that gray matter regions outside our selected ROIs were highly unlikely to produce correlations that were comparable to those found in early visual cortex. High correlations did not arise due to a bias in our voxel selection method, and could not be explained by overall signal-to-noise ratio, or number of voxels within each ROI. The ability of a given region to represent the physical shift did strongly predict variability in the size of the correlation effect in different participants, across the five functionally defined visual areas. This suggests that our experiment design may be biased against areas with weak retinotopic responses to physical stimuli. The receptive field size of retinotopic areas, as measured with fMRI, increases up the visual processing hierarchy (Harvey and Dumoulin, 2011). Later areas with larger receptive fields may be less able to differentiate the two physical shift locations, which would explain this pattern of results. The relationship between physical shift and effect size did not quite reach significance when computed for the 12 atlas-defined ROIs, so differences in receptive field size may not fully account for the lack of effect in later areas.

Even if our analysis favors early over later visual areas, the main result nonetheless reveals a neural correlate of shifts in perceived position in early visual areas. This is surprising in a number of ways. When compared directly, patterns of fMRI activity in early visual areas have been found to reflect physical stimulus position more strongly than perceived position, whereas in later areas like hMT+ and LOC perceived position was more predictive (Fischer et al., 2011). Similarly, Maus et al. (2013a) found evidence for a motion-dependent change in the neural representation of object position in areas V3A and hMT+, but no such evidence in early visual areas. Several psychophysical studies of motion-induced position shifts also provide some support for the preeminence of higher-level visual areas in representing perceived position. Motion-induced position shifts appear to follow global, rather than local, motion (Mussap and Prins, 2002; Hisakata and Murakami, 2009; Mather and Pavan, 2009; Rider et al., 2009), although recent evidence suggests that local motion also makes an important contribution to the effect (Kohler et al., 2015). High-level motion signals can cause a position shift, in the near-absence of low-level motion signals (Watanabe et al., 2003). The Flash Drag Effect can occur with anorthoscopic perception, when the real stimulus motion is reduced to zero (Watanabe et al., 2002), or even in the total absence of net motion energy in the image (Shim and Cavanagh, 2004). Finally, when motion signals arise from overlapping transparent surfaces, the flash grab effect is driven by the attended surface (Tse et al., 2011). Because many of these higher level motion phenomena have been shown to be mediated by hMT+ and other areas late in the visual processing stream, all of these findings lend further support to the hypothesis that information about perceived position is not available until later stages of visual analysis, with hMT+ as a strong candidate for the region in which motion exerts its influence over perceived position (McGraw et al., 2004).

Given the discrepancy between the present and past findings, what are we to make of the high correlations that we observe in early visual cortex? The first fMRI evidence for an effect of motion on representations of object position in early visual areas was provided by Whitney and colleagues (Whitney et al., 2003) who found that retinotopic patterns of activation were shifted in the opposite direction of the perceptual effect. Importantly, this effect could be reproduced under conditions that did not lead to an illusory position shift (Whitney et al., 2003), indicating that it may be a more general effect of motion signals on patterns of activity, independent of perceived location. Later publications have argued that patterns of activations were in fact not shifted at all, and that the effects were instead driven by changes in the spatial distribution of responses that were smaller than the spatial resolution of retinotopy (Liu et al., 2006). This effect could potentially be driven by neuronal response biases to motion in different directions (Liu et al., 2006), because the measured shifts in retinotopy in a given direction always co-occurred with motion in the opposite direction (Whitney et al., 2003; Wang et al., 2014). A recent, more comprehensive, investigation of these effects measured population receptive fields (pRF; Dumoulin and Wandell, 2008) from voxels in a wide range of retinotopic areas across visual cortex, presenting bi-directional motion inside the bar stimulus used to map the pRFs (Harvey and Dumoulin, 2016). They found direction- and speed-dependent changes in pRF size and eccentricity in all visual field maps examined, and that effects were approximately proportional to pRF sizes, suggesting that with the stimulus used, representations of visual space are influenced by motion in a similar way throughout the visual hierarchy (Harvey and Dumoulin, 2016). Harvey and Dumoulin (2016) were unable to distinguish the effect of individual motion directions, but based on the previous findings they proposed that pRF preferred positions were displaced against the direction of motion, producing the changes in size and, due to a differential effect of motion toward and away from fixation, eccentricity (Harvey and Dumoulin, 2016).

In our experiment, the stimuli were checkerboard patterns overlaid on bi-directionally moving patterns, and we correlated activations evoked by these stimuli with activations evoked by physically shifted checkerboards presented without motion. The only difference between our two motion-shift conditions lay in the timing of the checkerboard presentation within each TR, while the motion was identical between conditions. Because we used rotational motion, differential effects of motion toward or away from fixation cannot explain our results. Furthermore, any effect of motion on neuronal responses that is consistent with the findings discussed above, would predict that patterns of activation should be shifted or biased away from the physically shifted checkerboards that were matched to perceived shift in position. Therefore, we propose that the high correlations we find in early visual cortex comprise a novel effect that is distinct from the earlier findings in early visual cortex.

BOLD activity in early visual cortex, measured with fMRI, can be influenced by attention (Gandhi et al., 1999; Martínez et al., 1999; Somers et al., 1999; Kamitani and Tong, 2005). Experiments by Cavanagh and Anstis (2013) have suggested that the flash grab effect requires attention, so it is likely impossible to separate the effect from attention. Attention could potentially account for our results through downward attentional projections to early visual cortex at the perceived locations, that would in turn result in BOLD signal responses that shift with perceived location during motion-shift blocks. We note that participants were engaged in the fixation task at a comparable level throughout the experiment, and were not asked to attend to the checkerboard stimulus or to the moving background. Nevertheless, a moving background and a checkered flash can be assumed to automatically draw exogenous attention, which could mediate downward projections to early visual cortex that might match the perceived locations, potentially accounting for the correlations between physical and perceived activations. However, if the high correlations in early visual cortex were in fact due to attention-related downward projections, one might expect similarly high correlations in IPS areas that are directly linked to spatial attention (Saygin and Sereno, 2008), which we did not see. We acknowledge, however, that our present data alone are not sufficient to rule out an attentional account of motion-induced position shifts that could involve feedback connections to V1–V3.

There are some aspects of the literature that support a role for early visual cortex in representing perceived position. Importantly, Fischer et al. (2011) did find evidence that activity patterns in early visual cortex were influenced by perceived position, although physical position was a stronger predictor. The recent demonstration that motion can have widespread influence on activity in retinotopic cortex (Harvey and Dumoulin, 2016) also provides some plausibility to our findings, even if the two sets of findings may not be driven by the same mechanism. Recent psychophysical evidence has demonstrated that shifting the perceived position of a grating using the flash grab effect leads to a corresponding shift in the location of the tilt aftereffect elicited by the grating (Kosovicheva et al., 2012), corresponding to about 10% of the perceived shift. Neurophysiological studies have indicated that the tilt aftereffect is driven by adaptation of orientation-selective cells in V1 (Movshon and Lennie, 1979), so this result suggests that at least some aspect of the mechanism underlying motion-induced position shifts can be attributed to early visual cortex. We emphasize that the high correlations we see in early visual cortex could result from a similar partial representation of the perceived shift, as indicated by our effect size analysis. Additional support for an early visual cortex component to the motion-induced position shift comes from a recent EEG experiment (Hogendoorn et al., 2015), which used multivariate pattern classification to investigate the temporal dynamics of the flash grab effect. They found that representations of physically identical stimuli that were perceived at different positions due to motion could be recovered as early as 81 ms post-stimulus, and that these representations were similar to those evoked by stimuli that were in fact physically shifted (Hogendoorn et al., 2015). This result suggests that there may in fact be an early, feed-forward component to the effect, which could potentially arise in early visual cortex. Finally, the size of the flash grab effect might also be an important reason why we saw an effect in early visual cortex, while others did not. If the perceived position signal in V1 is weak, it might be necessary to use a stimulus that induces large differences in perceived position, such as the flash grab effect. Previous fMRI studies that investigated perceived position used illusory position shifts that were smaller than the ones we used (Fischer et al., 2011; Maus et al., 2013a), may have failed to detect effects in early visual cortex because signal failed to rise above the level of noise.

Traditionally, the striate cortex of V1 was believed to consist mostly of neurons with the classical receptive field properties first described by Hubel and Wiesel (1962). These neurons can represent information available in the retinal image with a high degree of accuracy, but can do little else (Fang et al., 2008a; Muckli, 2010). Multiple recent fMRI experiments, however, have demonstrated that early visual cortex can also play a role in representing emergent perceptual properties that are not present in the retinal image, in multiple different domains of visual perception. The outcome of size (Murray et al., 2006) and lightness (Boyaci et al., 2007) constancy operations, various types of filling-in (Meng et al., 2007; Hsieh and Tse, 2010), apparent motion (Muckli et al., 2005; Sterzer et al., 2006), conscious visibility under meta-contrast masking (Tse et al., 2005), and perceptual grouping (Murray et al., 2002; Fang et al., 2008b) all lead to differentiated processing in V1, effects that cannot be easily explained in terms of classical receptive field properties or local interactions. Many researchers have proposed that feedback from areas later in the visual processing stream modulates neural activity in V1 (Murray et al., 2002), and there is some evidence to support this hypothesis, at least for apparent motion (Wibral et al., 2009).

It is possible that coding of perceived position in V1 and V2 is indicative of a similar type of feedback that modulates position signal in V1 to reflect perceived, rather than physical position. Some versions of the motion-induced position shift, such as the Flash Drag Effect, arise as a result of feedback from hMT+ to V1 (Nishida and Johnston, 1999). TMS could potentially disrupt this feedback, although data from a later study have not directly supported this hypothesis (Maus et al., 2013b). The authors proposed that position shifts are in fact partially coded in V1, and that stimulus factors, as well as the retinotopic specificity of V1, can explain the absence of TMS effects in V1 (Maus et al., 2013b).

The fact that we found no evidence that hMT+ encodes perceived as opposed to retinal position poses a problem for the hypothesis that feedback signals from hMT+ give rise to the effects that we observe in early visual cortex. As mentioned earlier, one possibility is that our experiment design favors early visual areas that have smaller receptive fields. Another possibility is that information about perceived position does in fact exist in hMT+, but that BOLD responses in hMT+ voxels are at ceiling, because the same voxels respond to both the moving stimulus and to the checkerboard. In the Flash Grab Effect, the flash and moving stimulus overlap spatially, which is not the case for stimuli used in previous studies (Maus et al., 2013a). Because hMT+ voxels are highly sensitive to motion, position information may not be detectable in these voxels, when they respond to both the moving stimulus and the checkerboard. This could mean that although position information is available both in early visual cortex and hMT+, we cannot detect it in hMT+. The only evidence we see of perceived position encoding in late visual areas is a marginally significant effect in atlas-defined V3A. Future studies should test whether this is due to the specifics of our stimulus and/or experiment design.

In summary, we can draw the following conclusions from this first fMRI experiment using the Flash Grab Effect. When a briefly presented checkerboard stimulus undergoes an illusory position shift due to the Flash Grab Effect, patterns of activation in early visual cortex, most prominently in V1, but also in V2 and V3, resemble the patterns elicited by a physical stimulus shift. Although highly significant in V1, we did not see this effect in any late visual areas, and correlations elsewhere in the brain were also systematically lower than those seen in early visual cortex. This is the first demonstration that early visual cortex may play a role in motion-induced position shifts.

## Ethics statement

This study was carried out in accordance with the recommendations of The Committee for the Protection of Human Subjects, the Institutional Review Board at Dartmouth College, with written informed consent from all subjects. All subjects gave written informed consent in accordance with the Declaration of Helsinki. The protocol was approved by The Committee for the Protection of Human Subjects.

## Author contributions

PK, PC, and PT conceived and designed the experiment. PK generated the stimuli. PK collected and analyzed the fMRI data. PK, PC, and PT wrote the paper.

### Conflict of interest statement

The authors declare that the research was conducted in the absence of any commercial or financial relationships that could be construed as a potential conflict of interest.
